# Fifteen-year experience with the Bicarbon heart valve prosthesis in a single center

**DOI:** 10.1186/s13019-015-0294-x

**Published:** 2015-06-28

**Authors:** Yoshio Misawa, Arata Muraoka, Shin-ichi Ohki, Kei Aizawa, Koji Kawahito, Tsutomu Saito, Hirotaka Sato, Ippei Takazawa, Soki Kurumisawa, Hirohiko Akutsu, Akira Sugaya

**Affiliations:** Division of Cardiovascular Surgery, Department of Surgery, Jichi Medical University, 3311-1 Yakushiji, Shimotsuke, Tochigi 329-0498 Japan

**Keywords:** Bicarbon valve, Heart valve disease, Valve replacement, Valve-related complication, Bileaflet prosthetic valve, Heart valve surgery

## Abstract

**Background:**

The purpose of this study was to evaluate retrospectively the clinical performance of the Bicarbon valve (Sorin Biomedica Cardio, Saluggia, Italy) implanted at our center in Japan.

**Methods:**

Between January 1997 and December 2011, 415 patients in our institution were implanted with the Bicarbon valve. Nine of these recipients were excluded from the study because they had already undergone valve implantation and received a Bicarbon valve in a different position. The remaining patients were analyzed for evaluation of the postoperative clinical outcomes. Of the 406 patients (mean age 60.2 ± 11.7 years), 179 underwent aortic valve replacement (AVR), 149 mitral valve replacement (MVR), and 78 both aortic and mitral valve replacement (DVR).

**Results:**

There were 10 early deaths (2.5 %: 4 in the AVR group and 6 in the MVR group). Three hundred eighty-nine patients were followed up (95.8 % completeness of follow-up) with a mean follow-up of 6.6 ± 4.2 years overall (AVR 6.8 ± 4.2, MVR, 6.7 ± 4.4, and DVR 5.7 ± 3.4 years) and a cumulative follow-up of 2661 patient-years (1214, 1001, and 446 patient-years for AVR, MVR, and DVR, respectively). Ninety-nine patients died (3.7 % per patient-year: 22 valve-related and 77 valve-unrelated deaths). Survival at 10 years was 74.1 ± 4.0 % in the AVR group, 73.7 ± 4.2 % in the MVR group, and 61.0 ± 7.9 % in the DVR group. The linearized incidence of thromboembolic complications, bleeding complications, prosthetic valve endocarditis, paravalvular leaks, and sudden death in all patients was 0.5 %, 0.5 %, 0.2 %, 0.2 %, and 0.4 % per patient-year, respectively. The incidence of valve-related complications and reoperation was 1.6 % and 0.4 %, respectively. No other valve-related complications were observed.

**Conclusions:**

The Bicarbon prosthetic heart valve has shown excellent clinical results and is associated with a low incidence of valve-related complications.

## Background

Many types of tissue and mechanical heart valve prostheses are commercially available worldwide. Lifelong anticoagulant therapy is inevitable for patients with mechanical prosthetic valves, and those with tissue valves have higher risks of structural valve dysfunction than those with mechanical ones. The purpose of this study was to evaluate retrospectively the clinical performance of the Bicarbon valve (Sorin Biomedica Cardio, Saluggia, Italy) implanted at our center in Japan.

## Methods

This retrospective follow-up study has been approved by Bioethics Committee of Jichi Medical University (approval number: A12-116). Between January 1997 and December 2011, 415 patients have been implanted with 496 Bicarbon valves at our hospital. For this study, nine of these patients were excluded because they had previously received another type of valve in addition to the Bicarbon. A total of 179 patients underwent aortic valve replacement (AVR), 149 mitral valve replacement (MVR), and 78 both aortic and mitral valve replacement (DVR). There were 230 men and 176 women of mean age 60.7 ± 11.7 years, most of whom were older than 60 years (Table 1; 162 patients in the 7th decade, 100 in the 6th, and 79 in the 8th). Valve re-replacement surgery was performed in 18 patients (3.7 %). Rheumatic disease was the most frequent cause of valve disease, followed by myxomatous degeneration, calcific aortic stenosis, and endocarditis (Table 2). The range of implanted valves is shown in Table 3. There was frequent use of sizes 19, 21, and 23 in AVR, and sizes 27 and 29 in MVR. In DVR cases, size 19 slimline and size 21 fitline valves were mainly implanted at the aortic position. Concomitant procedures were shown in Table 4. Tricuspid valve annuloplasty, coronary artery bypass grafting, and replacement of the ascending aortic surgery including aortic root replacement are major concomitant interventions.

**Table 1:** Age distribution

Age classes	Overall (cases)	AVR (cases)	MVR (cases)	DVR (cases)
20 yrs≧	2	1	1	0
21–30 yrs	9	6	2	1
31–40 yrs	17	10	5	2
41–50 yrs	33	13	13	7
51–60 yrs	100	38	44	18
61–70 yrs	162	74	60	28
71–80 yrs	79	34	23	22
81–90 yrs	4	3	1	0

*AVR* aortic valve replacement, *MVR* mitral valve replacement, *DVR* aortic and mitral valve replacement, *yrs* years

**Table 2:** Etiology of valve diseases

Etiology	aortic valve (cases)	mmitral valve (cases)
aortic dissection	6	0
calcific stenosis	54	4
congenital malformation	28	4
infective endocarditis	22	29
ischemic disease	0	15
myxomatous degeneration	75	72
prosthetic valve dysfunction	3	9
rheumatic disease	63	87
others	7	4

**Table 3:** Distribution of implanted valve sizes

1) Aortic position		
size	AVR (cases)	DVR (cases)
17	5	5
19	52	27
21	62	36
23	44	10
25	16	0
2) Mitral position		
size	MVR (cases)	DVR (cases)
25	10	12
27	79	48
29	60	18

*AVR* aortic valve replacement, *MVR* mitral valve replacement, *DVR* aortic and mitral valve replacement

**Table 4:** Concomitant procedures

procedure	Overall (cases)	AVR (cases)	MVR (cases)	DVR (cases)
aortic surgery	41	38	2	1
coronary artery bypass grafting	51	24	19	8
maze procedure	28	2	15	11
tricuspid valve annuloplasty	58	4	35	19
others	14	10	2	2

*AVR* aortic valve replacement, *MVR* mitral valve replacement, *DVR* aortic and mitral valve replacement

The mid-term results of 105 patients with a Bicarbon valve as of the end of January 2000 and the prospective follow-up study of the same patients at the end of December 2005 have already been reported [1, 2]. Clinical data from additional patients was evaluated on the basis of mortality and morbidity analysis.

Operative procedures and postoperative management have been described in the previous study [1]. The international normalization ratio (INR) was controlled to maintain values between 1.8 and 3.3 for patients having MVR and DVR, and was between 1.3 and 1.8 for AVR patients before 2000. Since then, the INR has been between 1.8 and 3.0 for all patients. Postoperative anticoagulation therapy did not differ with regard to rhythm status. An antiplatelet agent such as dipyridamole or aspirin was added to the anticoagulation therapy.

As of the end of June 2012, 406 patients participated in the follow-up study through an office interview, personal phone call, or information from patients’ family physicians. Seventeen patients were lost to follow-up (95.8 % completeness of follow-up) with a mean follow-up of 6.6 ± 4.2 years overall (AVR, 6.8 ± 4.2 years; MVR, 6.7 ± 4.4 years; DVR, 5.7 ± 3.4 years) and a cumulative follow-up of 2661 patient-years (1214, 1001, and 446 patient-years for AVR, MVR, and DVR, respectively).

### Statistical analyses

Analyses were performed overall on the implanted population and stratified according to implant site (aortic, mitral, and both aortic and mitral). Morbidity analysis included all cardiovascular complications as defined by Edmunds et al. [3]. Mortality data (cumulative survival) and incidence of clinical adverse events (freedom from events) were analyzed using the Kaplan-Meier actuarial method and 95 % confidence interval (CI)) by implant site.

Valve-related complications included thrombosis, embolism, anticoagulant-related bleeding, endocarditis, and valve dysfunction, and are presented as linearized rates. For each linearized rate, upper confidence limits (95 % CI) are provided, according to the method reported by Grunkemeier and Anderson [4]. For continuous variables, descriptive statistics (mean, standard deviation, range) are provided. Qualitative variables are presented as number of patients and occurrence. All analyses were performed using StatView v.5 (SAS Institute Inc., Cary, NC, USA).

## Results

### Survival and clinical functional class

**Fig. 1:**
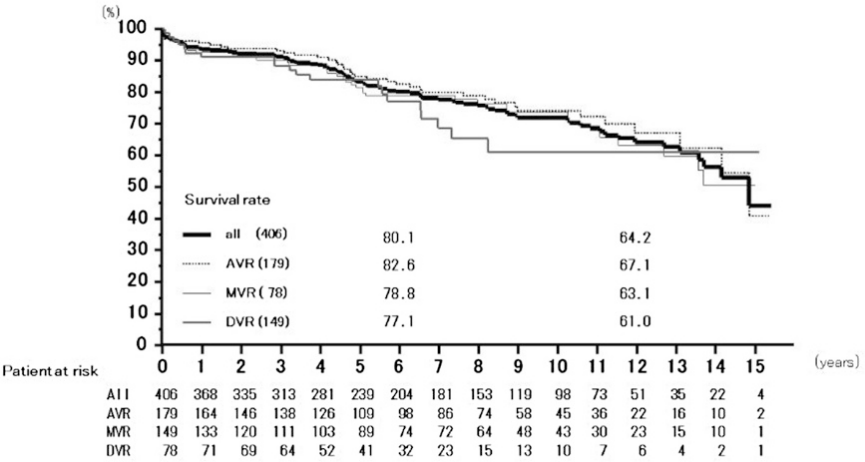
Survival from all death. Numerical values of the graph express numbers of patients at the time of follow-up. Abbreviations; All: all cases including AVR, MVR, and DVR cases; AVR: aortic valve replacement cases, MVR: mitral valve replacement cases; DVR: both aortic and mitral valve replacement cases

Ninety-nine patients died, including 10 hospital deaths (6 low cardiac output syndromes, 1 non-obstructive mesenteric infarction, 1 sepsis, 1 arrhythmia, and 1 aortic rupture). Late mortality consisted of 17 heart failures, 11 renal failures, 11 malignancies, 9 sudden deaths, 7 cerebral hemorrhages (including 4 subarachnoidal hemorrhages), 9 respiratory failures, 6 cerebral infarctions, 6 hepatic failures, 3 myocardial infarctions, 3 accidents, and 7 for other reasons. Survival rates at 6 years were 80.1 % (95 % CI: 75.9–84.5 %) overall, 82.6 % (76.6–89.0 %) for AVR, 78.8 % (71.9–86.3 %) for MVR, and 77.1 % (67.1–88.6 %) for DVR, and those at 12 years 64.2 % (57.6–71.6 %), 67.1 % (57.3–78.5 %), 63.1 % (52.8–75.4 %), and 61.0 % (47.3–78.6 %), respectively (Fig. 1).

**Fig. 2:**
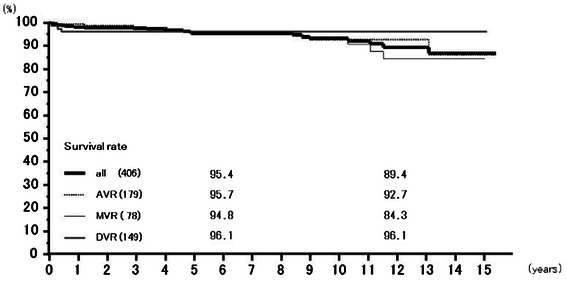
Survival from valve related death. Abbreviations; All: all cases including AVR, MVR, and DVR cases; AVR: aortic valve replacement cases, MVR: mitral valve replacement cases; DVR: both aortic and mitral valve replacement cases

**Fig. 3:**
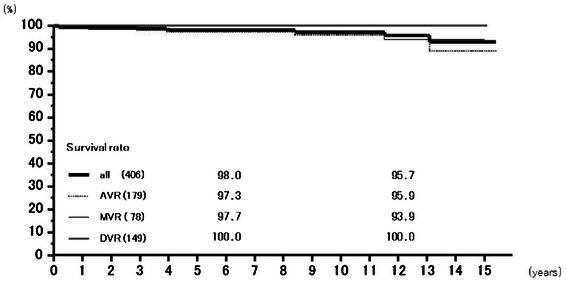
Freedom from sudden death. Abbreviations; All: all cases including AVR, MVR, and DVR cases; AVR: aortic valve replacement cases, MVR: mitral valve replacement cases; DVR: both aortic and mitral valve replacement cases

Valve-related death occurred in 9 patients in the AVR group, 10 in the MVR group, and 3 in the DVR group. Rates of freedom from valve-related death at 6 years were 95.4 % (93.2–97.8 %) overall, 95.7 % (92.4–99.2 %) for AVR, 94.8 % (90.8–99.0 %) for MVR, and 96.1 % (91.9–100.00 %) for DVR, and those at 12 years 89.4 % (84.3–94.9 %), 92.7 % (87.6–98.1 %), 84.3 % (74.2 − +95.7 %), and 96.1 % (91.9–100.00 %), respectively (Fig. 2). Valve-related death included 10 sudden death cases. Rates of freedom from sudden death at 6 years were 98.0 % (96.4–99.5 %) overall, 97.3 % (94.7–100.0 %) for AVR, 97.7 % (95.0–100.0 %) for MVR, and 100 % for DVR, and those at 12 years 95.7 % (92.1–99.4 %), 95.9 % (92.1–99.8 %), 93.9 % (86.6–100.0 %), and 100 %, respectively (Fig. 3)

Preoperatively, 66.6 % of the patients were in the New York Heart Association (NYHA) functional class III or IV, and 95.0 % of the surviving patients were in class I or II at follow-up.

### Valve-related complications

**Fig. 4:**
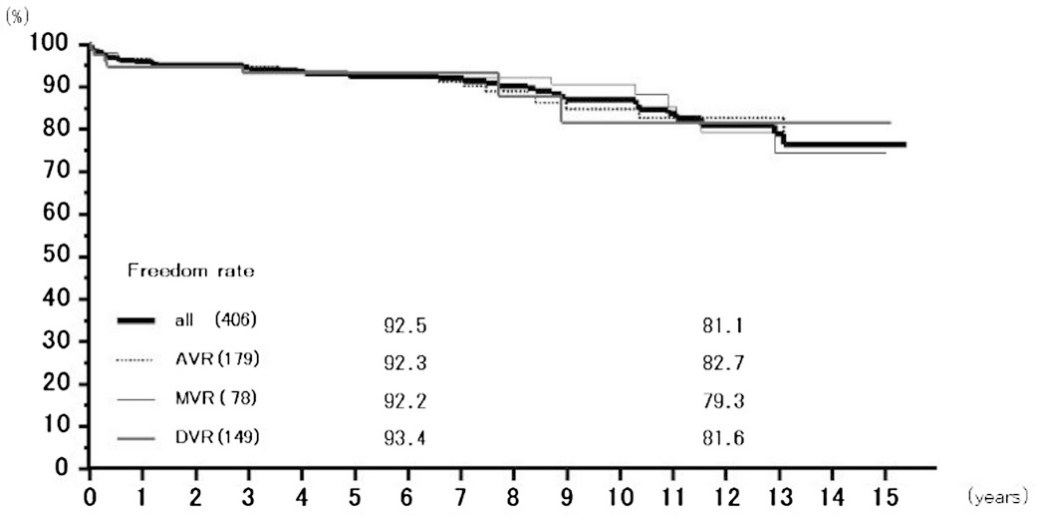
Freedom from all valve related complications. Abbreviations; All: all cases including AVR, MVR, and DVR cases; AVR: aortic valve replacement cases, MVR: mitral valve replacement cases; DVR: both aortic and mitral valve replacement cases

Forty-five valve-related complications were observed in the whole group, including 13 bleeding complications, 12 thromboembolisms, 5 endocarditis, and 5 paravalvular leakages without endocarditis. Ten patients with prosthetic valve endocarditis, paravalvular leakage or prosthetic valve thrombosis underwent reoperation. Rates of freedom from all valve-related complications at 6 years were 92.5 % (89.7–95.3 %) overall, 92.3 % (88.2–96.6 %) for AVR, 92.2 % (87.7–97.0 %) for MVR, and 93.4 % (87.9–99.2 %) for DVR, and at those at 12 years 81.1 % (75.0–87.8 %), 82.7 % (75.2–91.1 %), 79.3 % (68.6–91.7 %), and 81.6 % (67.0–99.4 %), respectively (Fig. 4).

**Fig. 5:**
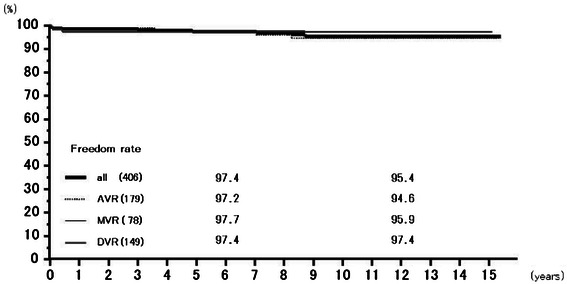
Freedom from thromboembolism. Abbreviations; All: all cases including AVR, MVR, and DVR cases; AVR: aortic valve replacement cases, MVR: mitral valve replacement cases; DVR: both aortic and mitral valve replacement cases

**Fig. 6:**
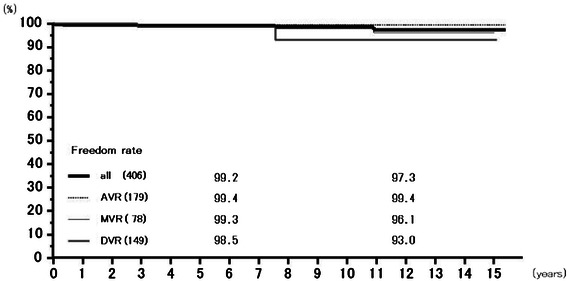
Freedom from prosthetic valve endocarditis. Abbreviations; All: all cases including AVR, MVR, and DVR cases; AVR: aortic valve replacement cases, MVR: mitral valve replacement cases; DVR: both aortic and mitral valve replacement cases

**Fig. 7:**
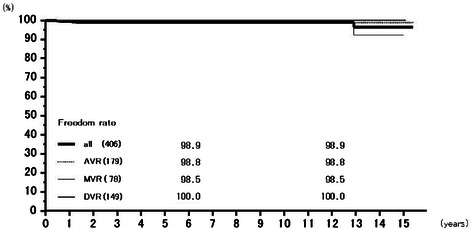
Freedom from paravalvular leakage. Abbreviations; All: all cases including AVR, MVR, and DVR cases; AVR: aortic valve replacement cases, MVR: mitral valve replacement cases; DVR: both aortic and mitral valve replacement cases

**Fig. 8:**
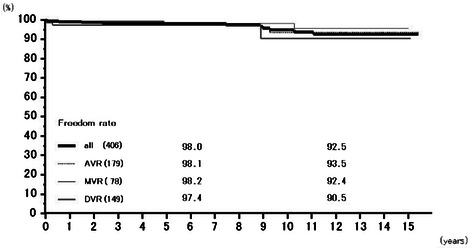
Freedom from hemorrhagic complications. Abbreviations; All: all cases including AVR, MVR, and DVR cases; AVR: aortic valve replacement cases, MVR: mitral valve replacement cases; DVR: both aortic and mitral valve replacement cases

**Fig. 9:**
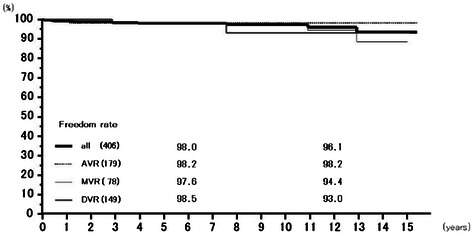
Freedom from reoperation. Abbreviations; All: all cases including AVR, MVR, and DVR cases; AVR: aortic valve replacement cases, MVR: mitral valve replacement cases; DVR: both aortic and mitral valve replacement cases

Rates of freedom from thromboembolism at 6 years were 97.4 % (95.7–99.1 %) overall, 97.2 % (94.5–100.0 %) for AVR, 97.7 % (95.2–100.0 %) for MVR, and 97.4 % (93.8–100.0 %) for DVR, and those at 12 years 95.4 % (92.7–98.3 %), 94.6 % (90.2–99.2 %), 95.9 % (91.8–100.0 %), and 97.4 % (93.8–100.0 %), respectively (Fig. 5). Freedom from prosthetic valve endocarditis at 12 years was 97.3 % (94.4–100.0 %) overall, 99.4 % (98.3–100.0 %) for AVR, 96.1 % (90.0–100.0 %) for MVR, and 93.0 % (82.8–100.0 %) for DVR (Fig. 6). Freedom from non-structural paravalvular leakages at 12 years was 98.9 % (97.9–100.0 %) overall, 98.8 % (97.2–100.0 %) for AVR, 98.5 % (96.5–100.0 %) for MVR, and 100 % for DVR (Fig. 7). Rates of freedom from hemorrhagic complications at 6 years were 98.0 % (96.5–99.5 %) overall, 98.1 % (96.0–100.0 %) for AVR, 98.2 % (95.7–100.0 %) for MVR, and 97.4 % (94.0–100.0 %) for DVR, and those at 12 years 92.5 % (88.0–97.3 %), 93.5 % (88.1–99.2 %), 92.4 % (84.65–100.0 %), and 90.5 % (77.9–100.0 %), respectively (Fig. 8). Rates of freedom from reoperation at 6 years were 98.0 % (96.6–99.5 %) overall, 98.2 % (96.3–100.0 %) for AVR, 97.6 % (94.9–100.0 %) for MVR, and 98.5 % (95.6–100.0 %) for DVR, and those at 12 years 96.1 % (93.1–99.3 %), 98.2 % (96.3–100.0 %), 94.4 % (88.1–100.0 %), and 93.0 % (82.8–100.0 %), respectively (Fig. 9). We experienced no structural prosthetic valve dysfunction.

### Linearized rates and upper confidence limits of events

The linearized rates and 95 % CI of each event overall were 0.5 % (0.3–0.9 %) per patient-year for thromboembolic events, 0.5 % (0.3–0.9 %) per patient-year for bleeding, 0.2 % (0.1–0.4 %) per patient-year for prosthetic endocarditis, 0.2 % (0.1–0.4 %) per patient-year for paravalvular leak, 0.4 % (0.2–0.7 %) per patient-year for sudden death, and 1.4 % (0.8–2.5 %) per patient-year for all valve-related complications.

Linearized rates of the AVR group were 0.5 % (95 % CI: 0.2–1.0 %) per patient-year for thromboembolic events, 0.5 % (0.2–1.0 %) per patient-year for bleeding, and 1.6 % (1.1–2.7 %) per patient-year for all valve-related complications. Those of the MVR group were 0.4 % (0.1–1.0 %) for thromboembolic events, 0.4 % (0.1–1.0 %) for bleeding, and 1.6 % (0.8–3.2 %) for all valve-related complications. Those of the DVR group were 0.5 % (0.1–1.6 %) for thromboembolic events, 0.7 % (0.2–1.9 %) for bleeding, and 1.6 % (0.6–3.5 %) for all valve-related complications.

## Discussion

Tissue valves are more likely than mechanical valves to be implanted in Japan, with 11,656 tissue and 5050 mechanical heart valves implanted in 2012 [5]. Prolonged durability of the tissue valves, an aging population, and an increasing number of plastic surgery techniques have led to a decrease in the use of mechanical valves. Nevertheless, mechanical valves appear to be a better option for some patients. At our institution we choose a mechanical valve for young adults and patients with end-stage renal disease because of the rapid structural dysfunction of tissue valves [6].

Excellent early and mid-term clinical results of the Bicarbon valve have been previously reported [1, 7–11]. We also reported early and mid-term results with the prosthetic valve in 2002 and 2007. Only long-term follow-up studies, however, will be sufficient enough to properly evaluate its clinical performance. Here we report our 15-year clinical experience with the Bicarbon valve.

With regard to single-center mid-term results, Goldsmith and colleagues reported in 1998 that the Bicarbon valve has a satisfactory clinical performance with low complication rates [7]. A 2004 multicenter study of the Bicarbon valve in Europe with a mean follow-up of 2.2 ± 1.5 years showed rates of 5 % early death and 4.4 % late death [8]. In addition, the linearized incidence of valve thrombosis was between 0.06 % and 0.69 % per patient-year among the AVR, MVR, and DVR groups, and that of embolic episodes was between 1.13 % and 2.14 % per patient-year. Bleeding complications occurred at a rate of 0.69–1.26 % per patient-year.

Other studies have shown that freedom from valve thrombosis at 7–9 years was between 97 % and 99.4 %, freedom from embolic episodes was between 64 % and 93 %, and freedom from bleeding complications was between 82 % and 98.6 % [9–11]. Actuarial analysis at 7–9 years showed an overall survival of between 63.9 % and 88 %.

Regarding long-term multicenter clinical results, Azarnoush and colleagues reported actuarial survival at 15 years of 61.4 % for AVR, 63.4 % for MVR, and 56.4 % for DVR [12]. They added that actuarial freedom from thromboembolism, anticoagulant-related hemorrhage, and endocarditis at 15 years was 88.8 %, 77.5 %, and 96.8 %, respectively, without any cases of structural failure of the prosthesis.

Long-term clinical experience with the St. Jude Medical and Carbomedics bileaflet mechanical valves for AVR and MVR have shown rates of thrombosis of between 0.73 % and 3.4 % per patient-year, and 10-year freedom from thrombosis of between 77 % and 94.2 % [12–21]. In addition, the rates of bleeding were between 0.52 % and 2.7 % per patient-year, and the 10-year freedom from bleeding ranged from 77 % to 96.4 %. Our previous reports concluded that the results obtained were similar to those for other mechanical valves associated with a low incidence of valve-related morbidity and mortality [1, 2].

Jeong and colleagues reported long-term clinical results with the ATS mechanical valve in 1382 consecutive patients, revealing that the survival rates at 12 years were 87.0 ± 3.8 % in the AVR group and 71.4 ± 6.3 % in the DVR group [22]. In addition, there was a higher cardiovascular event-free survival at 12 years in the AVR group than the DVR group (82.3 ± 4.7 % versus 65.1 ± 7.3 %). Another long-term study was conducted by Sezai, who reported bleeding events at a rate of 0.19 % and thromboembolic events of 0.44 % per patient-year in 231 cases over 15 years of observation [22].

In the present era, a bileaflet valve is more likely than a tilting disc valve to be implanted. In 2001 we revealed our 14-year experience with the Omnicarbon tilting disc valve in 168 consecutive patients of mean age 53 years [23]. Freedom from thromboembolism at 10 years was 94 % in the AVR group, 80 % in the MVR group, and 96 % in the DVR group, and that from hemorrhagic complications was 86 %, 92 %, and 100 %, respectively. The Ominicarbon valve showed good clinical performances similar to the Bicarbon valve. The increasing age of patients with a narrow aortic valve annulus and posterior leaflet preservation techniques in MVR have led us to choose bileaflet mechanical valves [24]. However, one must be aware of the possible risk of the valve becoming stuck because of the posterior orientation of the major orifice.

Although structural dysfunction has been overcome, valve-related complications after heart valve replacement with mechanical valves occur at unacceptable rates. Thromboembolic and hemorrhagic events related to anticoagulant therapy should be considered during lifelong follow-up. Non-structural prosthetic valve dysfunctions such as paravalvular leak and pannus ingrowth are also issues to be resolved [25].

The present study gives additional evidence of low rates of valve-related complications after Bicarbon valve implantation. Our anticoagulation therapy is associated with an antiplatelet agent. Patients with INR >3.0 sometimes develop lethal bleeding complications, so we maintain the INR between 1.8 and 3.0. The rate of thromboembolic events in this study is excellent and the rates of bleeding complications are also acceptable. We confirm that anticoagulation for patients with a mechanical valve should be implemented in association with an antiplatelet agent. Postoperative NYHA functional improvement was satisfactory in all groups. Low cardiac output syndrome led to lethal complications in those patients with advanced stage IV NYHA classification. To obtain better clinical outcomes, an operation before advanced functional class is reached should be considered, and more intensive perioperative management may be mandatory for patients with heart failure that is far advanced.

### Limitations

Many patients have been followed by their family physicians for several months after surgery for reasons of patients’ convenience. Some visit our outpatient clinic once or twice a year, while others do not. We sometimes experience poorly controlled anticoagulant therapy among such patients, which might lead to thromboembolic and bleeding complications. Unfortunately, we were unable to evaluate the INR of some patients suffering from thromboembolic and/or bleeding events. In addition, deaths from unknown causes are included in the category of sudden death, meaning that the rate of cases of sudden death may be higher than its true incidence.

## Conclusions

This single-center study of a 15-year follow-up of the Bicarbon prosthetic heart valve shows excellent clinical results associated with a low incidence of valve-related mortality and morbidity.
